# COVID-19 risk factors and outcomes in individuals with stiff person syndrome spectrum disorders before and after omicron

**DOI:** 10.1186/s13023-024-03357-w

**Published:** 2024-09-27

**Authors:** Hanyeh Afshar, Alexandra Simpson, Elena Taylor, Ashley Miles, Herbert R. Chen, Scott D. Newsome

**Affiliations:** Johns Hopkins University School of Medicine, Baltimore, MD USA

**Keywords:** Stiff person syndrome, SPSD, COVID-19, Neurology, Vaccination

## Abstract

**Background:**

Stiff person syndrome spectrum disorders (SPSD) are rare, disabling disorders of the nervous system that are associated with risk factors for Coronavirus disease 2019 (COVID-19). However, limited data exist on the overall impact of COVID-19 on SPSD.

**Methods:**

Patients with SPSD and COVID-19 who are followed at Johns Hopkins SPS Center were included. Demographics and SPSD characteristics along with COVID-19-specific data were recorded.

**Results:**

Thirty-five cases of SPSD with COVID-19 cases were reported during the study time period. Mean age of the cohort was 56 (SD ± 10) and most were female (66.7%). Eighty percent of the COVID-19 cases were confirmed with testing, and the rest were highly suggestive of COVID-19. COVID-19 comorbidities among patients were hypertension (n = 6), diabetes (n = 6), obesity (n = 5), and cardiovascular disease (n = 4). The majority of participants were on immune therapies and/or benzodiazepines. Out of the cases reported, only 2 required hospitalization, both of whom had diabetes, and one was on immunosuppressive therapy. The majority of cases were post-full-vaccination cases. Fever was the most common COVID-19-associated symptom. Transient neurological symptoms were also reported.

**Conclusion:**

Risk factors for developing severe COVID-19 in SPSD appear to be the same as historical data in the general population. Importantly, COVID-19 did not appear to be associated with worsening SPSD post-COVID-19. Vaccination may have played a role in preventing severe cases of COVID-19.

## Introduction

Confounding factors might make individuals with stiff person syndrome spectrum disorders (SPSD) appear to be at a higher risk for adverse outcomes for Coronavirus disease 2019 (COVID-19) compared to the general population. SPSD are rare, debilitating disorders of the nervous system, that are primarily characterized by progressive muscle rigidity and intermittent muscle spasms [1, 2]. Even though the exact pathophysiology of SPSD is still unknown, the syndrome is widely accepted as an autoimmune disorder, with the muscle stiffness/spasms and other symptoms resulting from the impairment of the γ-aminobutyric acid (GABA)-ergic signaling by anti-glutamic acid decarboxylase (antiGAD65) or other associated antibodies [1].

Clinical manifestations of COVID-19 can vary greatly in the general population, ranging from asymptomatic to severe, and with fever, cough, shortness of breath, anosmia, myalgia or fatigue, and sputum production being the most common symptoms (28.5–88.7%); other symptoms can include sore throat (11%), headache (8%), and gastrointestinal problems, such as diarrhea (6.1%) [3]. Studies show that individuals are at a higher risk of getting severely ill with Severe acute respiratory syndrome coronavirus 2 (SARS-CoV-2) if they suffer from certain underlying conditions, such as diabetes, cardiovascular disease, or ambulatory impairment [3–5]. A large percentage of patient with SPSD can have type 1 diabetes mellitus (T1DM) and experience  ambulatory dysfunction ranging from requiring an assistive device to walk to becoming wheelchair-bound or worse [2]. Besides the co-presence of these health issues  with SPSD, the type of therapies that are commonly used in the management of these disorders may also inadvertently put patients at a higher risk for severe COVID-19 outcomes. For instance, immunosuppressant therapies (IST), such as rituximab and mycophenolate mofetil, are often used in patients who do not respond to symptomatic and/or first-line immune therapies. Studies on COVID-19 outcomes in multiple sclerosis (MS) patients as well as patients with other health conditions like rheumatoid arthritis have demonstrated that B-cell-depleting therapies can put patients at a higher risk for severe COVID-19 outcomes like hospitalization and need for mechanical ventilation [6–8]. Similarly, chronic use of benzodiazepines (BZD), a group of medications commonly used for managing SPSD symptoms, has been shown to be associated with a higher risk of hospitalization from COVID-19 [9].

Omicron is one of the newer variants of SARS-CoV-2 that is shown to be less likely to cause severe infections and hospitalization regardless of COVID-19 vaccination status but more likely to break through vaccination protection than the earlier variants [10, 11]. Considering the presence of COVID-19 risk factors in the SPSD population and the different incidence rates and trends of COVID-19 seen with the Omicron variant, the present study aims to explore the overall impact of COVID-19 on SPSD along with assessing if risk factors exist for severe COVID-19 in SPSD and if COVID-19 outcomes in SPSD patients vary for the Omicron variant compared to the previously-identified variants of SARS-CoV-2.

## Methods

### Study design and participants

This observational study investigated the impact of COVID-19 on patients with SPSD from March 2020 through December 2022. All participants are part of the IRB-approved Johns Hopkins Stiff Person Syndrome (SPS) Center longitudinal study cohort. Data for this study was collected and entered into our Research Electronic Data Capture (REDCap) SPS database, which consists of comprehensive, detailed demographic data, and clinical information pertinent to SPSD, such as SPS phenotype, antibody status, burden of disease, date of symptom onset and diagnosis, amongst other data. Specific to the current study, patients with SPSD were contacted via phone call or electronically via email and inquired if they had a history of COVID-19 or had experienced symptoms highly suggestive of COVID-19 and were willing to participate in our study and provide additional details about their experience with COVID-19 over a telephone interview. Those who expressed interest in participating in our study were then scheduled for phone interviews. One patient was interviewed via Zoom due to residing out of the United States. For data quality and accuracy, data that were not available in the patient’s medical records were obtained via interview. The COVID-19-related data were obtained through medical records and via self-report by the patients during the interviews. Hence, data was only collected for the individuals who had expressed both a history of COVID-19 or symptoms and interest in participating in our study. The demographic and clinical characteristics collected during the interview that were relevant to COVID-19 (some cross-validation with medical records) included the types of therapies patients were on for SPSD at the time of COVID-19, the medical comorbidities patients had (e.g., diabetes, obesity, cancer, etc.), tobacco use history (classified as never, past, current, unknown), current DMT and symptomatic therapies used for SPSD at the time of SARS-CoV-2 infection, COVID-19 symptoms, duration of their COVID-19 symptoms, COVID-19 testing modality, COVID-19 treatments, time period of COVID-19, COVID-19 vaccination history, and overall outcomes. No data was obtained on the prevalence of COVID-19 in the SPSD cohort.

### Statistical analysis

Data captured into REDCap case report forms (CRF) was first analyzed and organized using REDCap data analysis feature. The data analyzed by REDCap was then exported into Excel for further statistical analysis. Descriptive statistics were performed to obtain the mean and standard deviation (SD) for the continuous data. Given that the cases recorded had occurred in two different countries, namely the United States and South Africa, post-Omicron was defined as occurring on or after November 23rd, 2021 for cases that occurred in South Africa, and on or after December 1st, 2021 for cases that occurred in the United States to accommodate for the timing difference in the introduction of the Omicron variant in each country [12, 13]. Requiring hospitalization was used as an indicator of severe COVID-19 illness. The categorical data were then analyzed using Excel COUNTIFS Function and Excel FREQUENCY function. PivotChart was used for data visualization.

## Results

### Demographics

**Fig. 1:**
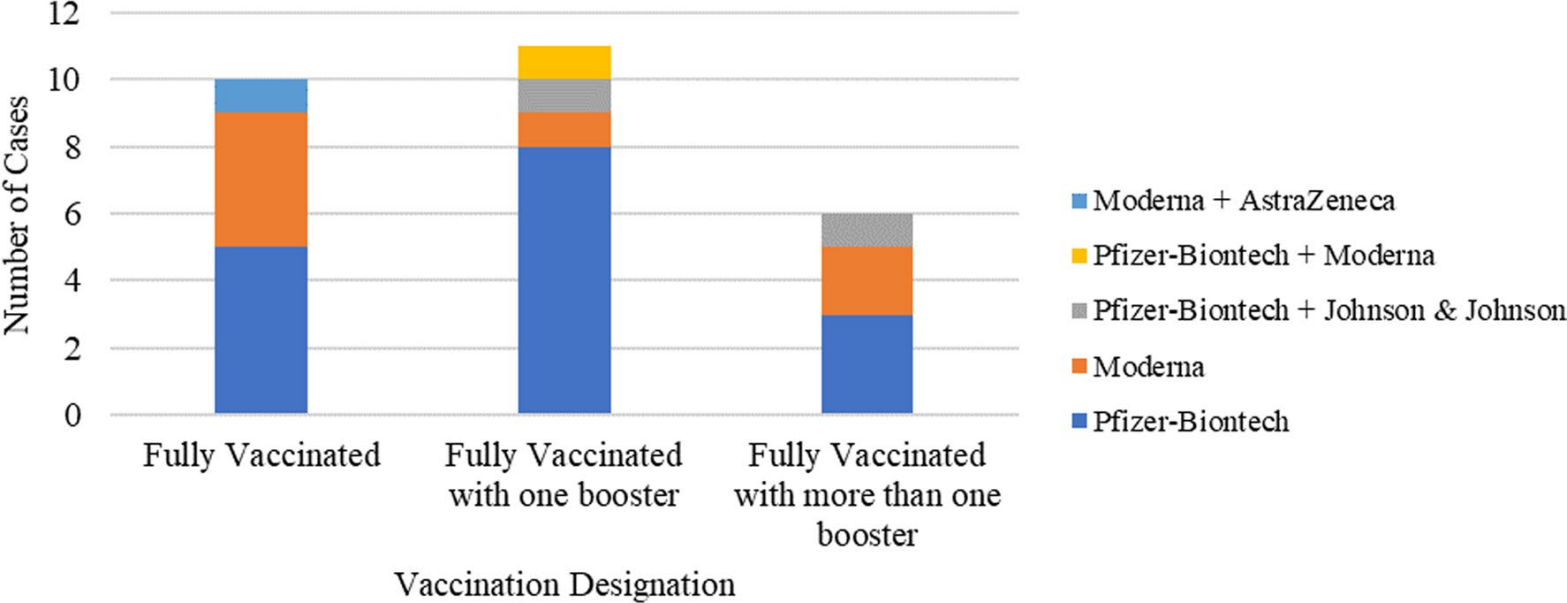
Vaccination status in post-full-vaccination cases

At the time of the study, Johns Hopkins SPSD cohort included 244 individuals, of whom 35 individuals reported a history of COVID-19 or were highly suspicious to have COVID-19 based on symptoms. We were able to interview and collect data from 27, including 9 males and 18 females. There were 21 White, 3 Black or African Americans, 2 Asians (including 1 self-identifying as Filipino), and 1 self-identifying as Indian. The participants had a mean age (± SD) of 43 (± 11.3) years at the time of SPS symptom onset. Twenty-two individuals had a diagnosis of classic SPS, and five had SPS-plus. There were 24 individuals with a positive serum anti-GAD65 antibody, two of whom also had positive anti-glycine receptors. Three individuals did not have any SPSD-associated antibodies. Past anti-amphiphysin testing results were available for 25 individuals, all of which were negative. Given that 7 individuals had experienced COVID-19 more than once, a total number of 35 cases of COVID-19 were recorded. Eighty percent of the cases were reported by participants as confirmed with testing, including 11 cases confirmed with PCR, 14 cases confirmed with rapid antigen tests, 1 case confirmed with serologic testing, and 1 case with method of testing being unknown. Total cases recorded consisted of 8 pre-Omicron cases, 26 post-Omicron cases, and 1 case with an unknown date of onset. Individuals involved in 22 of the total cases had their anti-GAD65 antibody titer measured within one year prior to the infection date with a mean anti-GAD65 antibody titer of 213,000 IU/mL (range, 0–1,570,000 IU/mL). Seventy-seven percent of the total cases recorded had occurred in individuals who were fully vaccinated against COVID-19 (Fig. 1).

Twenty-three cases out of the 24 post-full-vaccination cases confirmed with testing were post-Omicron cases. The mean age (± SD) at the time of COVID-19 was 56 (± 10) years. The most frequently reported COVID-19 comorbidities by the participants were diabetes (n = 6) and hypertension (n = 6) followed by cardiovascular disease (n = 4) and chronic lung disease (n = 3), including asthma (n = 2). Other COVID-19 comorbidities reported were chronic liver disease (n = 1) and having a BMI falling within the obese (n = 5) or morbidly obese range (n = 1). There were two patients with a remote history of cancer (1 individual with past medical history of papillary carcinoma of the thyroid and 1 individual with past endometrial cancer) who underwent surgery but not chemotherapy or radiation. Other COVID-19 risk factors reported by the interviewees besides the comorbidities were current tobacco use (n = 4), mobility impairment  (5 individuals using a unilateral assistive device, 1 individual requiring a bilateral assistive device, and 1 individual being wheelchair-dependent), and receiving SPSD therapies that have been shown to be a risk factor for experiencing poor COVID-19 outcomes, namely immunosuppressant therapies or benzodiazepines, as demonstrated in Table 1.

**Table 1:** Types of therapy used for stiff person syndrome

Type of immunosuppressant therapy	Number of individuals
Intravenous immunoglobulin	13
Rituximab	7
Azathioprine	1

Type of symptomatic therapy	Number of individuals
Diazepam	20
Clonazepam	12
Lorazepam	1
Baclofen	14
Methocarbamol	2
Tizanidine	3
Pregabalin	3
Botulinum toxin	3
Unknown^a^	2
Other^b^	4

^a^Participants were unsure about taking symptomatic therapy at the time of COVID-19

^b^Other symptomatic therapy agents used by patients were trihexyphenidyl, carbamazepine, amitriptyline, neuropathy cream, over-the-counter non-steroidal anti-inflammatory drugs, vitamins, and supplements

### COVID-19 symptoms, treatments, and outcomes

**Fig. 2:**
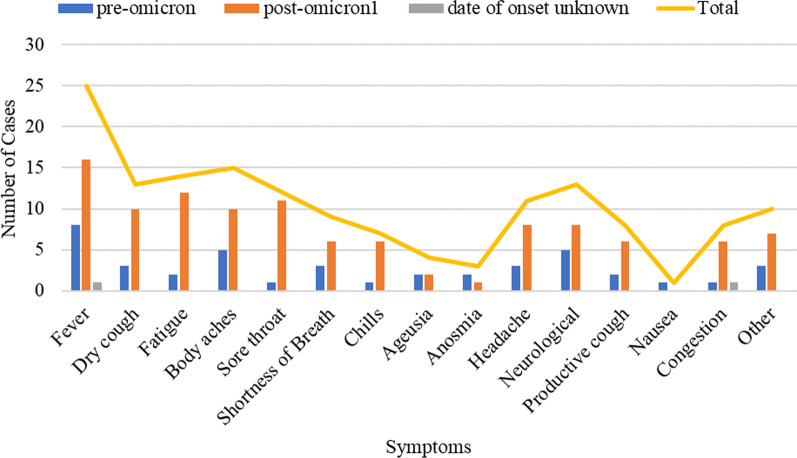
COVID-19 symptoms among the studied cases. Post-Omicron period was defined as occurring on or after November 23rd, 2021 for the case that occurred in South Africa, and on or after December 1st, 2021 for cases that occurred in the United States

**Fig. 3:**
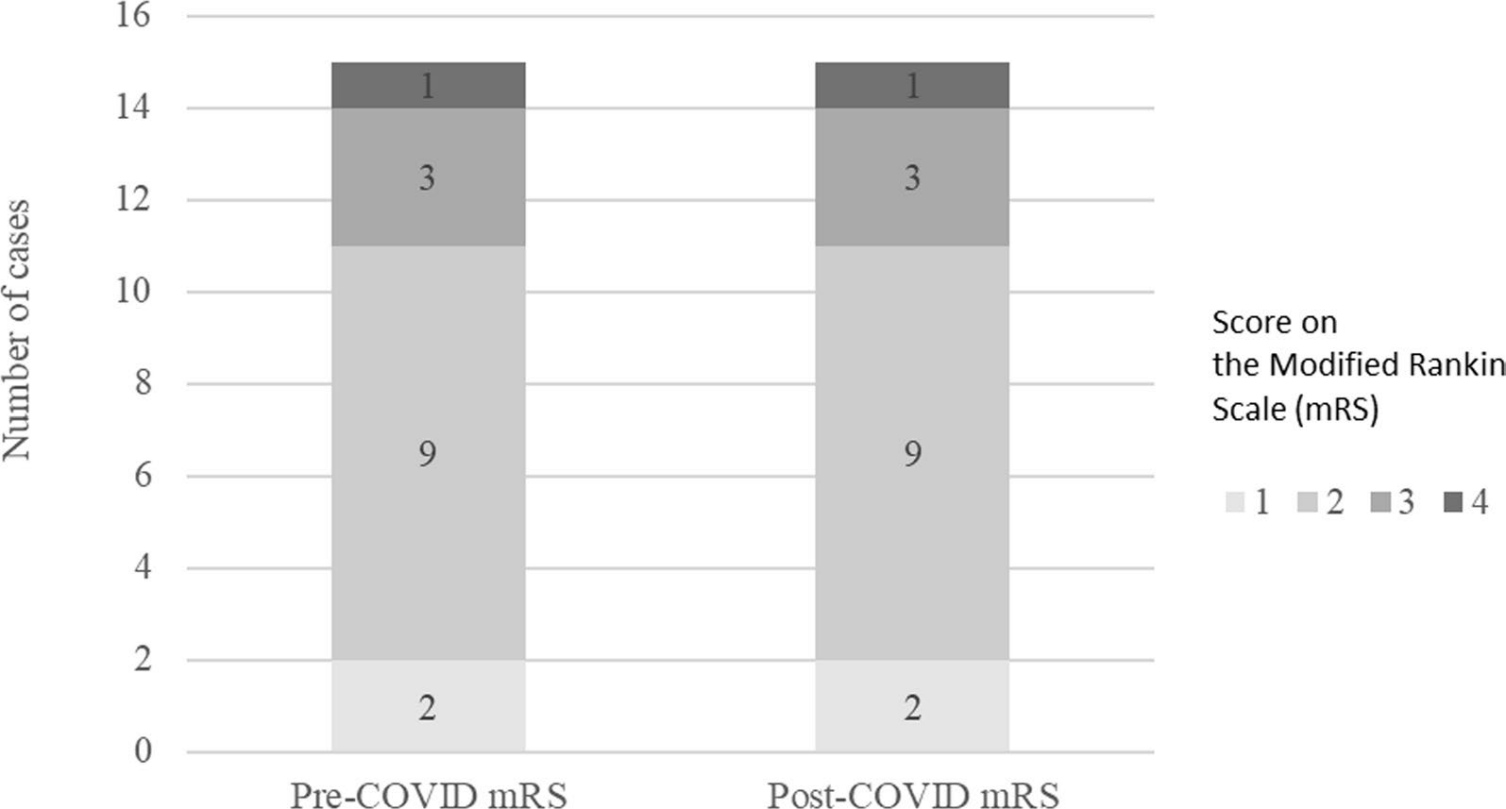
Scores on the Modified Rankin Scale (mRS) before and after infection with COVID-19

Fever, dry cough, fatigue, body aches, sore throat, headache, and neurological symptoms were the most common symptoms among the cases (Fig. 2). Fever was the most common symptom reported for both the post-full-vaccination cases as well as the cases involving individuals not fully vaccinated. Neurological symptoms mainly included exacerbated SPSD symptoms, namely more severe, frequent, or diffuse spasms (n = 8) and stiffness (n = 3). Other neurological symptoms reported were incoordination (n = 1) and cognitive dysfunction (n = 1). Three individuals reported only experiencing severe spasms specifically at the time of fever, one of whom also reported experiencing neuropathy at the time of fever. Despite these subjective reports, there was no change in the Modified Rankin Scale (mRS) following infection with COVID-19 (median mRS = 2, IQR 2–2) for the 15 cases for which we had both the pre-and post-COVID mRS scores within one year of the SARS-CoV-2 infection date (Fig. 3).

**Fig. 4:**
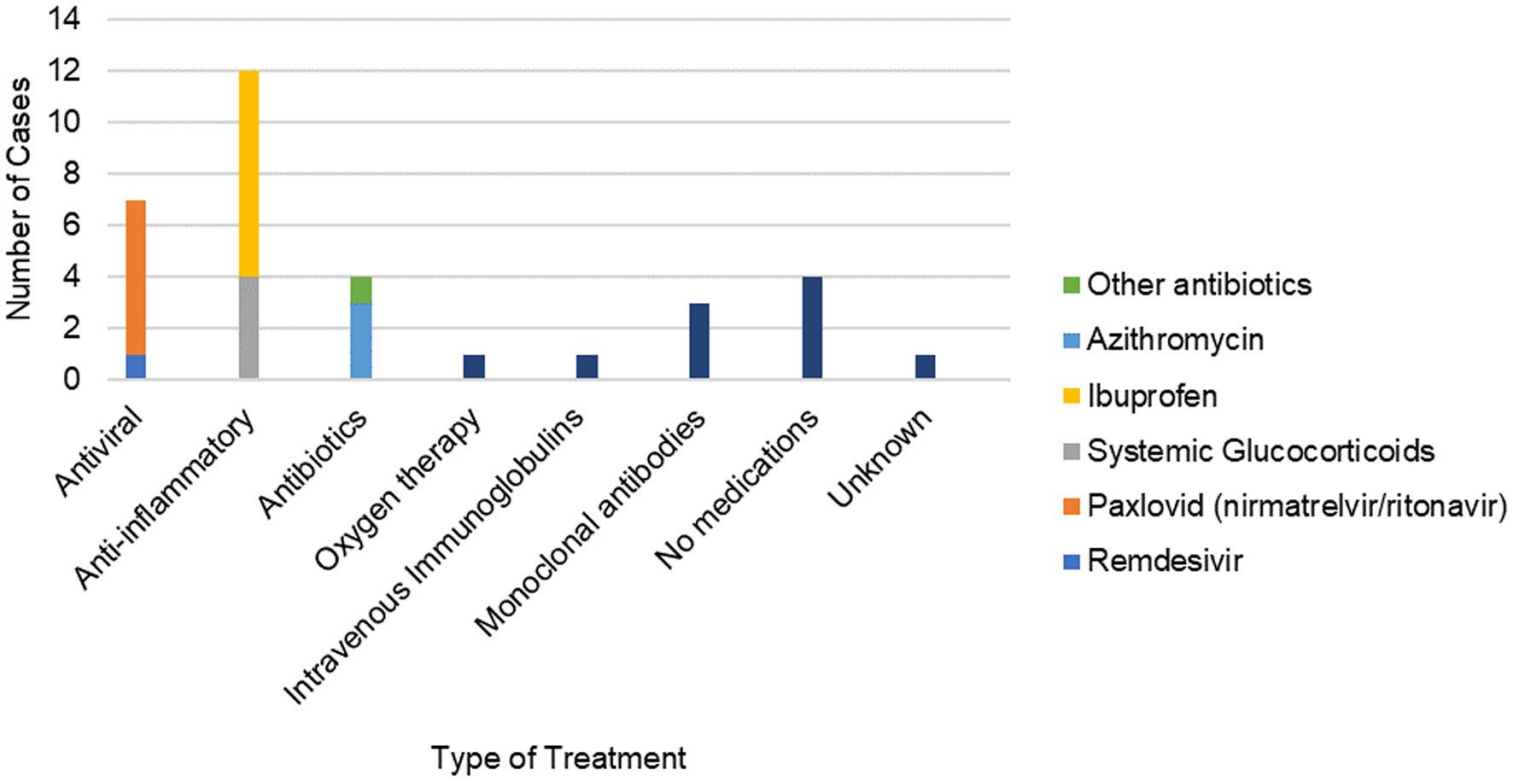
Type of treatment used for COVID-19 cases studied

As shown in Fig. 4, anti-inflammatory drugs (n = 12), namely ibuprofen (n = 8) and systemic glucocorticoids (n = 4), were the most common types of treatments used for COVID-19, followed by antiviral medications (n = 7), particularly Paxlovid (n = 6), and then antibiotics (n = 4), particularly azithromycin (n = 3).

As described in Table 2, there were two severe COVID-19 cases requiring hospitalization, both of which were also post-full-vaccination cases. One of the severe cases was diagnosed outside of the United States in Kenya.

**Table 2:** Severe COVID-19 cases

Case	Gender	Age	IST	BZD	Comorbidities	Vaccine type	ICU admission	Treatment	Outcome
1	Female	46	IVIG	Yes	Asthma, diabetes	Moderna + AstraZeneca	Yes, with invasive ventilation	Unknown	Recovered
2	Male	49	Rituximab	Yes	Diabetes	Pfizer + J&J (Boosted)	No	Remdesivir Systemic Glucocorticoids Oxygen therapy Ibuprofen Virtussin Benzonatate	Recovered

Abbreviation: IST, immunosuppressant therapy; IVIG, intravenous immunoglobulin; BZD, benzodiazepines; J&J, Johnson & Johnson

## Discussion

Based on the clinical presentations, treatments, and outcomes of COVID-19, in this study, we were able to demonstrate that COVID-19 affects individuals with SPSD in a similar way that it affects the general population as seen in historical data [3]. In detail, 94% of the COVID-19 cases reported in our study were mild-to-moderate with symptoms that are common with COVID-19, such as fever, cough, sore throat, body aches, and fatigue. These mild-to-moderate cases were treated with various therapies including antiviral medications, anti-inflammatory medications, or no medications. There were two severe cases requiring hospitalization, both of which involved risk factors (diabetes, IST, BZD) shown by previous studies for severe COVID-19 outcomes. To illustrate, a study of the COVID-19 outcomes in individuals with MS demonstrated that MS patients on B-cell-depleting therapies are at a higher risk for hospitalization and need for mechanical ventilation [7]. This has also been seen with other conditions like rheumatoid arthritis [8]. Similarly, chronic use of BZD has been shown to be associated with severe COVID-19 outcomes [9]. Although the presence of COVID-19 risk factors in our severe cases supports previous studies by demonstrating comorbidities and receiving IST or BZD as COVID-19 risk factors, the small sample size and lack of a control group limit full interpretability as it relates to SPSD as a separate risk as well as determining to what degree each of these risk factors contributes to individuals developing severe COVID-19 in SPSD.

Another COVID-19 risk factor existing among our study participants was mobility impairment. Several studies on COVID-19 risk factors in MS have shown that the degree of ambulatory impairment is highly associated with COVID-19 severity in people with MS [4, 5]. Only one person reported to be wheelchair-bound whose COVID-19 case was mild-to-moderate (mRS = 3). One of our severe cases involved a patient with mobility impairment using a unilateral ambulatory assistive device; no recent pre-COVID mRS was available for this case. There were four other participants using the same type of assistive device as this participant, all of whom experienced mild-to-moderate COVID-19. Even though all of our participants with ambulatory dysfunction, except for one of them, experienced mild-to-moderate COVID-19, the small sample size and lack of a control group do not allow us to contradict the findings of previous studies regarding ambulatory dysfunction being a risk factor for adverse COVID-19 outcomes.

It is also worth noting that six of the mild-to-moderate cases were treated with Paxlovid, which is a prescription medicine used in adults to reduce the risk of developing COVID-19 complications [14]. Paxlovid seemed to be well-tolerated and might have prevented more severe illness in those treated. Besides Paxlovid, vaccination may also have contributed to the low number of severe cases in our study as 77% of our cases were post-full-vaccination cases, although not knowing the exact vaccination timing with respect to the infection onset prevents us from determining whether vaccine-induced immunity has been involved in preventing poor COVID-19 outcomes in our post-full-vaccination cases.

Our study findings are consistent with the findings of previous studies regarding the vulnerability of the COVID-19 vaccines against the Omicron variant. That is, research has shown that the Omicron variant is more likely to pass the vaccine protection and infect vaccinated individuals compared to the older variants [10, 11]. Similarly, in our study, 23 out of the 24 post-full-vaccination cases confirmed with testing, including one of the severe cases, were post-Omicron cases. Although this finding further supports the idea that the Omicron variant might be a variant of concern when it comes to COVID-19 vaccine effectiveness, due to the small sample size used in our study and the exact timing of vaccination with respect to the infection date being unknown, we are limited in our interpretability of the Omicron variant as a variant of concern in terms of COVID-19 severity and its ability to break through the vaccine-induced immunity.

In conclusion, our study showed that COVID-19 risk factors existing in SPSD population are similar to those present in the general population. Risk factors of immunosuppressant therapies, benzodiazepines, and comorbidities like diabetes, existed in both our severe cases as well as the mild-to-moderate ones. Considering this and our study limitations, namely the small size and lack of a control group, we are unable to fully interpret how much each of these risk factors has contributed to the overall COVID-19 experience of our patients.

Symptoms experienced by the SPSD patients in our study were symptoms characteristics of COVID-19 (e.g., fever, body aches, fatigue, etc.) except for neurological symptoms, which manifested as amplified SPSD symptoms or other neurological symptoms, such as cognitive dysfunction. Nonetheless, increased SPSD symptoms in the setting of COVID-19 were transient and did not worsen SPSD as highlighted by the stable mRS post-COVID-19. Overall, the fact that 77% of our cases involved patients who were fully vaccinated and that 94% of the cases in our study were mild-to-moderate despite the high prevalence of COVID-19 risk factors among our study participants adds to the importance of considering preventative measures, such as vaccination, in patients with risk factors for severe COVID-19.

## Data Availability

Anonymized data will be shared by the corresponding author upon reasonable request.

## Abbreviations

SPSD: Stiff person syndrome spectrum disorders
COVID-19: Coronavirus disease 2019
SARS-CoV-2: Severe acute respiratory syndrome coronavirus 2
GABA: γ-Aminobutyric acid
antiGAD65: Anti-glutamic acid decarboxylase
T1DM: Type 1 diabetes mellitus
IST: Immunosuppressant therapy
MS: Multiple sclerosis
SPS: Stiff person syndrome
REDCap: Research electronic data capture
CRF: Case report form
SD: Standard deviation
IVIG: Intravenous immunoglobulin
mRS: Modified rankin scale
BZD: Benzodiazepines
J&J: Johnson & Johnson
